# Sensor-Assisted Analysis of Autonomic and Cerebrovascular Dysregulation following Concussion in an Individual with a History of Ten Concussions: A Case Study

**DOI:** 10.3390/s24134404

**Published:** 2024-07-07

**Authors:** Courtney M. Kennedy, Joel S. Burma, Jonathan D. Smirl

**Affiliations:** 1 Cerebrovascular Concussion Laboratory, Faculty of Kinesiology, University of Calgary, Calgary, AB T2N 1N4, Canada; jonathan.smirl@ucalgary.ca; 2 Sport Injury Prevention Research Centre, Faculty of Kinesiology, University of Calgary, Calgary, AB T2N 1N4, Canada; 3 Human Performance Laboratory, Faculty of Kinesiology, University of Calgary, Calgary, AB T2N 1N4, Canada; 4 Libin Cardiovascular Institute of Alberta, University of Calgary, Calgary, AB T2N 1N4, Canada; 5 Alberta Children’s Hospital Research Institute, University of Calgary, Calgary, AB T2N 1N4, Canada; 6 Hotchkiss Brain Institute, University of Calgary, Calgary, AB T2N 1N4, Canada; 7 Integrated Concussion Research Program, University of Calgary, Calgary, AB T2N 1N4, Canada

**Keywords:** autonomic function, dynamic cerebral autoregulation, neurovascular coupling, physiological recovery, bipolar disorder, attention deficit hyperactivity disorder, cerebral blood flow velocity

## Abstract

Introduction: Concussion is known to cause transient autonomic and cerebrovascular dysregulation that generally recovers; however, few studies have focused on individuals with an extensive concussion history. Method: The case was a 26-year-old male with a history of 10 concussions, diagnosed for bipolar type II disorder, mild attention-deficit hyperactivity disorder, and a history of migraines/headaches. The case was medicated with Valproic Acid and Escitalopram. Sensor-based baseline data were collected within six months of his injury and on days 1–5, 10, and 14 post-injury. Symptom reporting, heart rate variability (HRV), neurovascular coupling (NVC), and dynamic cerebral autoregulation (dCA) assessments were completed using numerous biomedical devices (i.e., transcranial Doppler ultrasound, 3-lead electrocardiography, finger photoplethysmography). Results: Total symptom and symptom severity scores were higher for the first-week post-injury, with physical and emotional symptoms being the most impacted. The NVC response showed lowered activation in the first three days post-injury, while autonomic (HRV) and autoregulation (dCA) were impaired across all testing visits occurring in the first 14 days following his concussion. Conclusions: Despite symptom resolution, the case demonstrated ongoing autonomic and autoregulatory dysfunction. Larger samples examining individuals with an extensive history of concussion are warranted to understand the chronic physiological changes that occur following cumulative concussions through biosensing devices.

## 1. Introduction

Concussions are a prevalent form of traumatic brain injury (TBI) [1] wherein trauma to cerebral structures often manifests as a transient disturbance in neurological function [2,3]. Clinical recovery from concussion is characterized by the return of physical, somatic, and/or cognitive signs and symptoms to prior injury baseline levels [1] albeit physiological recovery may occur beyond the window of clinical recovery [4,5]. Concussive symptoms typically resolve in 2–4 weeks in adolescents and 1–2 weeks in adults [6]. However, 10–30% of individuals may experience persistent post-concussion symptoms (PPCS), defined as experiencing concussion symptoms greater than the aforementioned adolescent and adult recovery thresholds [7]. This injury has been shown to induce metabolic and physiological changes within the brain, including those to dynamic cerebral autoregulation (dCA) [5,8,9], neurovascular coupling [10,11,12], and autonomic nervous system (ANS) function [13,14,15,16,17,18]. However, the extent to which cumulative concussions result in long-term impairments to these physiological processes is unknown. 

Previous studies have demonstrated that initial symptom burden is associated with the duration it takes to become asymptomatic (i.e., usually, clinical recovery is defined as the return of symptoms to baseline levels or a symptom severity score below 7 across three consecutive days) [6,19]. Thus, quantifying cardiovascular and cerebrovascular parameters immediately following an insult and tracking them in a detailed and systematic manner will provide valuable insight into the physiological underpinnings of concussion trajectories. Moreover, establishing clinical recovery is obfuscated by the heterogenous effect of concussion on the homeostatic mechanisms regulating brain function, complicating injury management and rehabilitation. Finally, it is known that various psychological/social factors will influence symptom recovery duration (e.g., mental health status, learning disorders, sex, etc.) [6,20,21,22,23,24,25,26].

As such, this case report study sought to quantify ANS and cerebral hemodynamic parameters across the initial 14 days following concussion in an individual with numerous risk factors for prolonged recovery. With the augmented concern of long-term cognitive ramifications associated with repetitive head trauma, these findings will help us to understand the cerebrovascular and cardiovascular cumulative impacts in a younger individual. Further, this case study addresses two of the ten top questions from the perspectives of clinicians, caregivers, and patients [27].

## 2. Case and Methods

The case of a 26-year-old male who sustained his 11th concussion by hitting his head on the frame of a sports utility vehicle is described. Information surrounding the date of injury, mechanisms of injury, and duration of recovery of the other 10 injuries are detailed in Table 1. Recovery was defined as the resolution of symptoms at or below 7 for three consecutive days, consistent with the previous literature [19]. All concussions were diagnosed by a trained physician and/or healthcare provider according to current guidelines for each injury time period [1,28]. Of importance, this individual has numerous known factors that are associated with prolonged recovery: diagnosis of type II bipolar disorder [26]; mild attention-deficit hyperactivity disorder (ADHD) [20]; extensive history of concussion (HOC), with half of these requiring greater than two weeks to experience recovery/symptom resolution [21]; and history of migraines/headaches [6]. One population-based study noted the highest risk of PPCS is for individuals with bipolar disorder [26], denoting the importance of the case study. The case was medicated for bipolar disorder type II with 250 mg of valproic acid and 15 mmHg of escitalopram. Ethical approval for this study was given by the Conjoint Health Research Ethics Board at the University of Calgary (REB: 21-1517).

It is the authors’ understanding that the data from this case report represent the most in-depth and most repeated assessments of dCA and autonomic testing ever reported in the field in an individual with an extensive history of concussion. Due to the time commitments associated with this level of physiological testing and the rarity of individuals with 10+ concussions, a larger cohort has not been feasible to collect at this level of resolution during the acute recovery stages following a concussion. All testing sessions occurred within the Cerebrovascular Concussion Laboratory at the University of Calgary. This included testing on days 1, 2, 3, 4, 5, 10, and 14 post-injury. Testing sessions were additionally planned for days 21 and 28; however, these were not possible as the host institution ceased all on-campus testing due to COVID-19 pandemic restrictions. Baseline data for all measures were collected for this individual in the six months preceding his injury. For each visit, the individual was subjected to transcranial Doppler ultrasound (TCD; DWL USA, Inc., San Juan Capistrano, CA, USA), beat-to-beat blood pressure monitoring (Finometer NOVA; Finapres Medical Systems, Amsterdam, The Netherlands), capnography (ML206; AD Instruments), and electrocardiography (FE 231 BioAmp; AD Instruments, Colorado Springs, CO, USA). The right middle cerebral artery (MCA) and left posterior artery (PCA) were insonated via TCD with two 2 MHz probes placed over the transtemporal windows [29]. A Finapres NOVA quantified blood pressure via finger photoplethysmography and corrected for the height of the heart. An inline gas analyzer was used to quantify the partial pressures of carbon dioxide and oxygen. All data were collected and time-synchronized using commercially available software (LabChart Pro Version 8, AD Instruments) at 1000 Hz. Prior to all testing sessions, the participant refrained from caffeine, exercise, smoking, alcohol, cannabis, and vaping for a minimum of 12 h [30]. 

At the start of the testing battery, the individual completed the symptom evaluation portion of the Sport Concussion Assessment Tool 5 (SCAT5) [31], as well as the overall condition via the graded 11-point visual analog scale [32]. He then subsequently completed five minutes of quiet sitting where heart rate variability (HRV) was measured [33]. Neurovascular coupling (NVC) was assessed using a complex visual-scene search “Where’s Waldo?” during eight cycles of 20 s eyes closed followed by 40 s eyes open while searching for the characters in the “Waldo Universe” [34,35,36,37]. HRV was quantified a second time within the upright orthostatic position [38]. Following this, five minutes of repeated squat-stand maneuvers (SSMs) were then performed in a randomized order to assess dynamic cerebral autoregulation (dCA) at frequencies of 0.05 and 0.10 Hz [39,40]. At the end of the testing session, his collective symptoms were recorded a second time using the SCAT5 Symptom Evaluation [31]. Symptom reports were computed as total symptom score and symptom severity score. Commercially available software (Ensemble-R V1.0.42, R&D Canvas, Wellington, New Zealand) was used to process HRV and dCA variables. HRV metrics were quantified within the time and frequency domains, with fast Fourier transformation being used to compute the latter. These metrics included heart rate, standard deviation of average normal–normal intervals (SDNN), root mean square of successive intervals (RMSSD), proportion of consecutive RR intervals that differ by more than 50 milliseconds (pNN50), relative low frequency (LF), and relative high frequency (HF), and LF/HF. The relative LF was calculated within 0.04–0.15 Hz [38,41,42,43], while the HF was calculated within 0.15–0.40 Hz [38]. NVC outcome metrics included baseline cerebral blood velocity (CBv) [44] during the 5 s eyes closed, peak CBv achieved during task engagement, the relative increase from baseline to peak, and the area-under-the-curve during the first 30 s of task engagement (AUC30) [34,35,36]. Transfer function analysis estimates were used to assess dCA, including coherence, phase, gain, and normalized gain, at both point-estimates of interest (i.e., 0.05 and 0.10 Hz) [39,40].

## 3. Results

**Figure 1:**
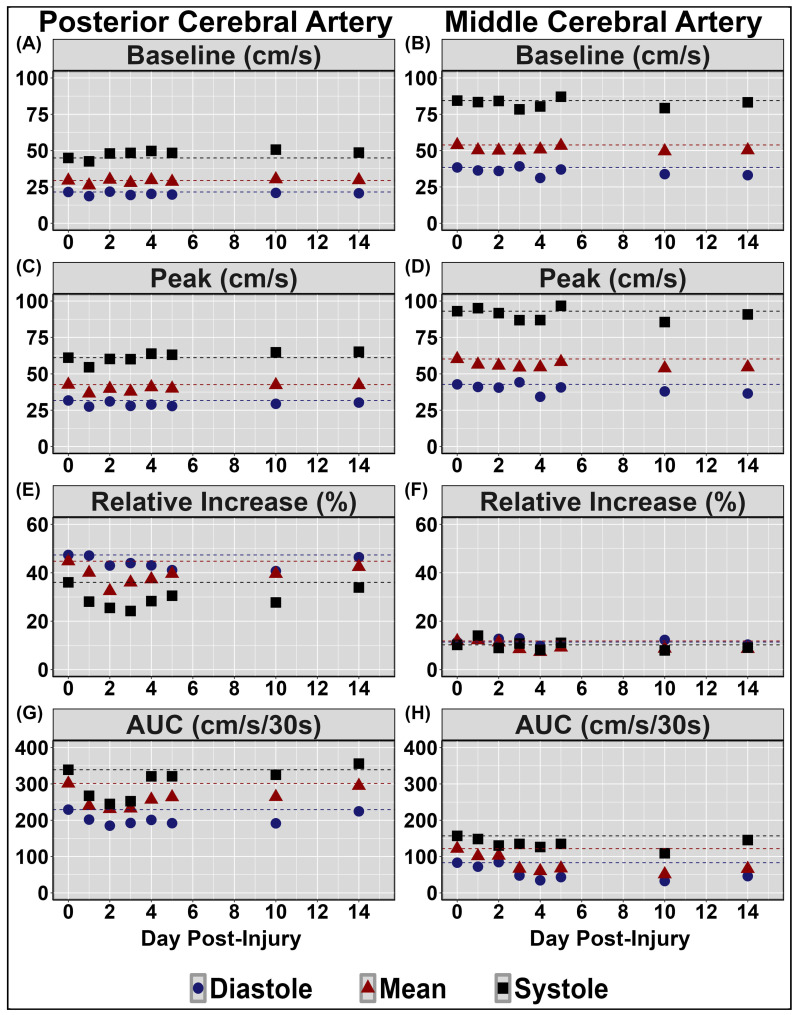
Neurovascular coupling results during a complex visual scene search (“Where’s Waldo?”). Centimetres per second (cm/s); percent (%); area under the curve (AUC). Units for all measures are displayed in the facet titles, in the posterior (**left** panel) and middle (**right** panel) cerebral arteries (PCA and MCA, respectively). Negligible change in CBFv observed throughout testing within the PCA at baseline, quantified during 5-seconds eyes closed, (panel (**A**)) and peak task engagement (panel (**C**)); and within the MVA at baseline (panel (**B**)) and peak (panel (**D**)). Note the substantial reductions across days 1–3 in AUC30 within the PCA, the primary region associated with the initial processing of the raw visual information associated with the scene-search task (panel (**G**)), but not the MCA (panel (**H**)), associated with motor and somatosensory function. The blunted relative increase from baseline to peak observed during systole in the posterior aspect did not return to pre-injury levels until day 14 (panel (**E**)), however no changes were observed within the MCA (panel (**F**)).

**Figure 2:**
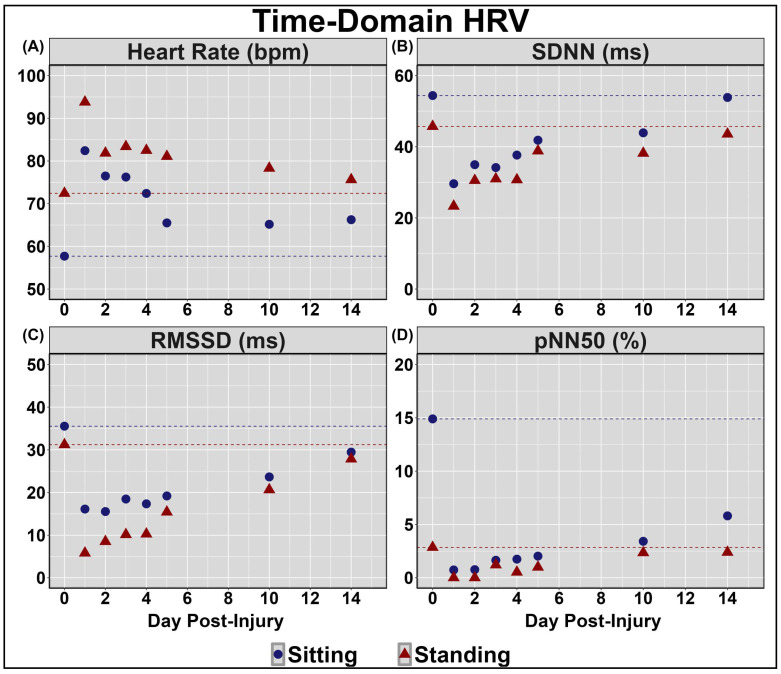
Time-domain heart rate variability (HRV) metrics collected during quiet sitting and standing periods. Standard deviation of average normal–normal intervals (SDNN); root mean square of successive intervals (RMSSD); proportion of consecutive RR intervals that differ by more than 50 ms (pNN50); beats per minute (bpm); milliseconds (ms); percent (%). Units for all measures are displayed in the facet titles. Note the substantial elevations in heart rate during both seated and standing periods that extended for the entire 14-day duration of testing (panel (**A**)), as well as the reductions in SDNN (panel (**B**)), RMSSD (panel (**C**)), and pNN50 (panel (**D**)) under both seated and standing conditions that were also present immediately following the injury, along with the consistent return towards baseline levels for these autonomic regulation metrics across the 14-day testing period.

**Figure 3:**
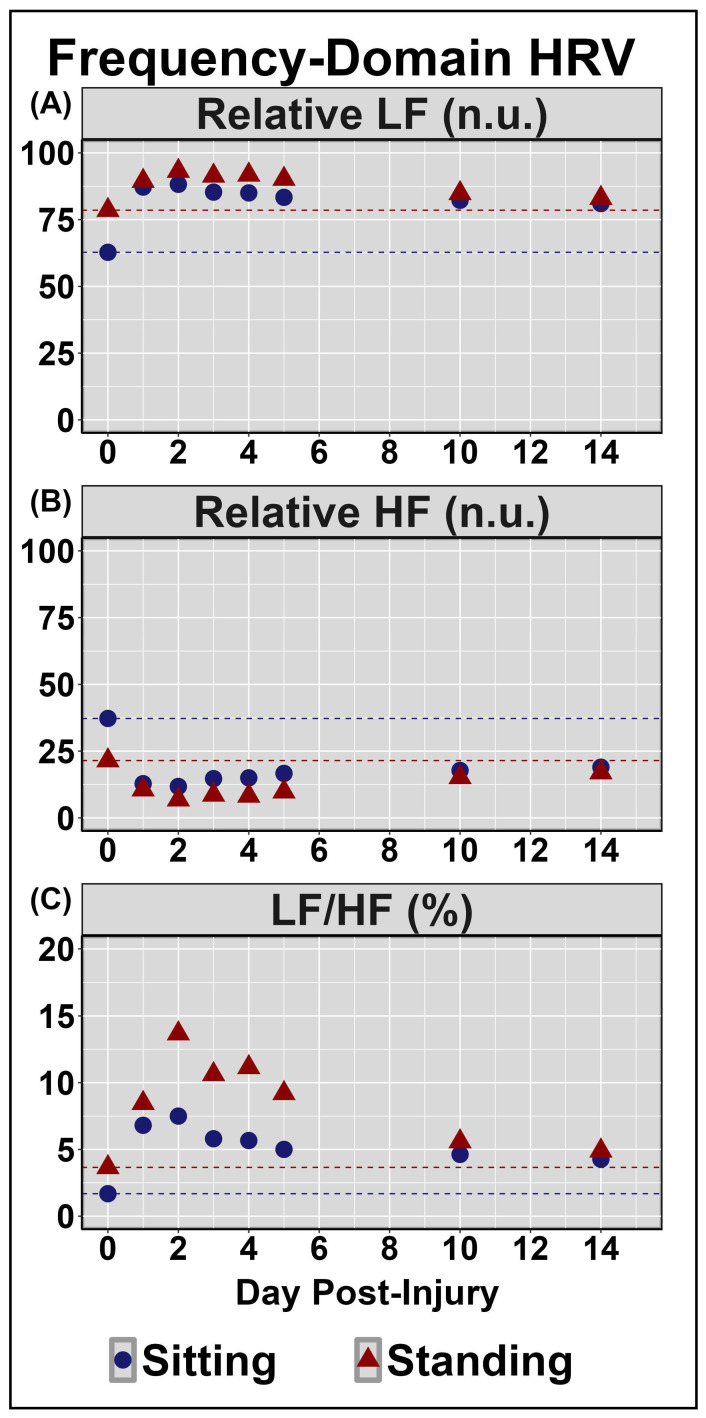
Frequency-domain heart rate variability (HRV) metrics collected during quiet sitting and standing periods. Low frequency (LF); high frequency (HF); normalized units (n.u.); percent (%). Units for all measures are displayed in the facet titles. Note the elevations in the relative LF (panel (**A**)) and LF/HF ratio (panel (**C**)) and the reductions in HF (panel (**B**)) across the 14-day testing period in this case.

**Figure 4:**
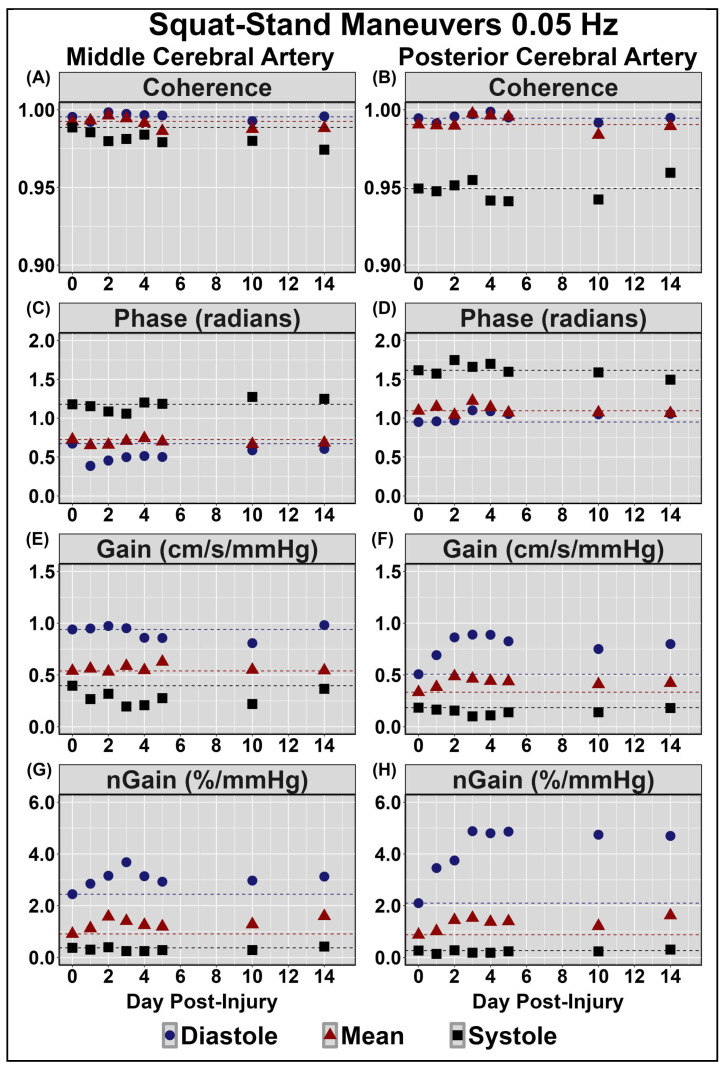
Dynamic cerebral autoregulation assessments via squat–stand maneuvers at 0.05 Hz in the middle (**left** panel) and posterior (**right** panel) cerebral arteries (MCA and PCA, respectively). Normalized gain (nGain); centimetres (cm); seconds (s); millimetres of mercury (mmHg); percent (%). Units for all measures are displayed in the facet titles. Negligible change observed in MCA coherence (panel (**A**)) and gain (panel **(E**)); and in PCA coherence (panel (**B**)) and phase (panel (**D**)). Note the slight reductions in diastolic middle cerebral artery (MCA) phase (panel (**C**)); elevations in nGain (panel (**G**)); and elevations in diastolic posterior cerebral artery (PCA) gain (panel (**F**)) and nGain (panel (**H**)).

**Figure 5:**
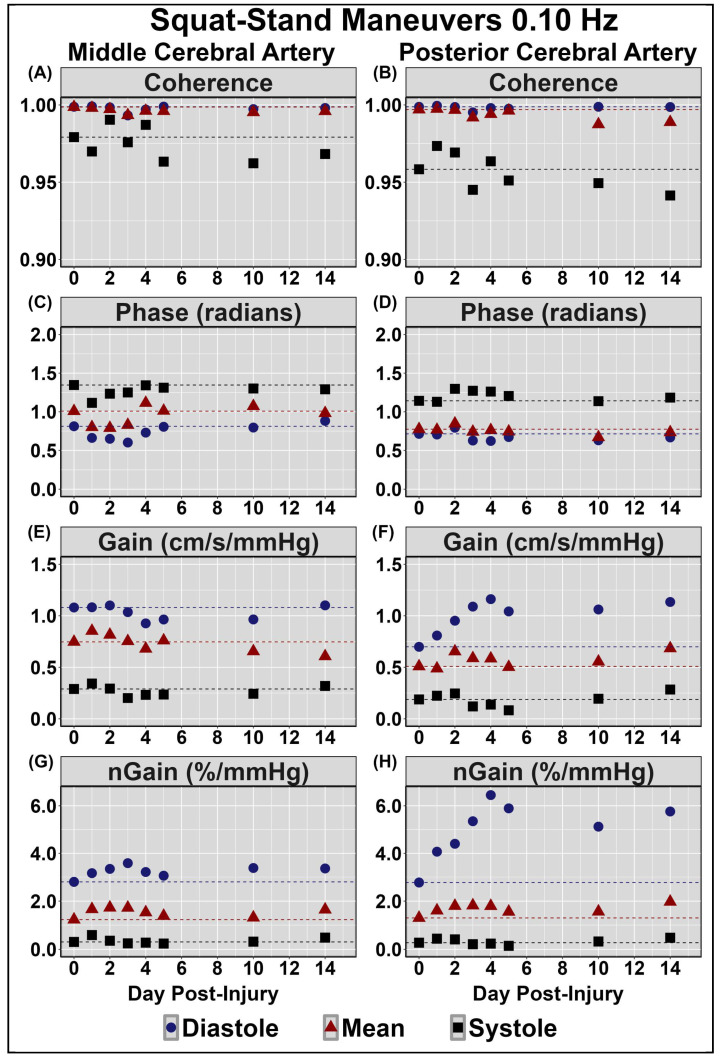
Dynamic cerebral autoregulation assessments via squat-stand maneuvers at 0.10 Hz in the middle (**left** panel) and posterior (**right** panel) cerebral arteries (MCA and PCA, respectively). Normalized gain (nGain); centimetres (cm); seconds (s); millimetres of mercury (mmHg); percent (%). Units for all measures are displayed in the facet titles. Negligible change observed in MCA coherence (panel (**A**)) and gain (panel (**E**)); and in PCA coherence (panel (**B**)) and phase (panel (**D**)). Note the slight reductions in diastolic and mean MCA phase (panel (**C**)) and elevations in nGain (panel (**G**)) and the elevations in diastolic PCA gain (panel (**F**)) and nGain (panel (**H**)).

Table 2 displays symptom reporting via the SCAT5 and overall condition. Figure 1 demonstrates the NVC coupling response assessed across the cardiac cycle over the 14 days post-injury. Minimal deviations were noted to baseline and peak CBv; however, PCAv AUC30 displayed the largest reductions days 1–3 post-injury, primarily within mean and systole (Figure 1). Moreover, the relative percent increase in PCAv was somewhat lower within systole. Figure 2 and Figure 3 display HRV metrics within the time and frequency domains, respectively. Heart rate was elevated during both sitting and standing rest paradigms, which translated to reductions in time-domain HRV metrics (Figure 2). The case also displayed a greater relative LF and subsequent LF/HF ratio (Figure 3). Collectively, the HRV changes indicate that the case had a greater degree of sympathetic activation and reduced vagal tone over the 14 days post-concussion (Figure 2 and Figure 3). Finally, during the SSMs, minimal deviation was noted in coherence metrics (Figure 4 and Figure 5). Both gain and normalized gain metrics were elevated across the 14 days in PCA at both 0.05 and 0.10 Hz, while phase reductions were noted within the MCA at both point-estimates of interest (Figure 4 and Figure 5). Collectively this is indicative of impaired dCA (Figure 4 and Figure 5). 

## 4. Discussion

The current case study examined the time course of cerebrovascular and cardiovascular parameters in a unique case with a history of 10 concussions, bipolar disorder, and ADHD (i.e., risk factors associated with prolonged recovery). There is an ever-growing concern about the long-term ramifications surrounding repetitive head trauma, of which the current case report highlights the acute physiological changes that occurred in an individual with a considerable history of concussion. Key findings were (1) symptoms were returned to baseline levels at 10 days post-injury; (2) the NVC response was attenuated for the first 3 days following injury; (3) a higher degree of sympathetic activation and reduced vagal tone was observed across the entire two-week span of testing post-injury; and (4) dCA assessed via cyclical 30–50 mmHg swings in blood pressure also demonstrated signs of impairment throughout this initial 2 week period following the injury. Collectively, these results indicate that ANS function and CBv regulation were impacted following concussion. While the duration of impairment differed between metrics, several factors persisted beyond clinical recovery. Findings from the current case study are congruent with the previous literature reporting a disconnect between clinical and physiological recovery of concussion [4], as overall condition and SCAT5 scores were reduced by day 7, although impairments in dCA and autonomic metrics persisted for 14 days post-injury. Nonetheless, given the a priori definition of concussion (i.e., seven or fewer symptom severity across three consecutive days), the case would not have reached clinical recovery until around day 14 or slightly thereafter. 

Results indicated that the case experienced multiple physiological disruptions following the concussive injury; that is, the NVC response was acutely altered following a concussive injury, as evidenced by the increased reductions in AUC30 observed on days 1–3 post-injury (Figure 1). This reduction may be attributed to the disruptions in neuronal signalling and axonal function associated with concussive injuries [10]. The neurometabolic cascade of concussion results in ionic flux and glutamate release, triggering hyperglycolysis and, subsequently, the depletion of energy reserves in order to restore ionic and cellular homeostasis [2,3]. An uncoupling between energy supply and demand is expected, as previous literature has reported reductions in cerebral blood flow (CBF) during this period of augmented metabolic demand [2,3,45,46,47,48]. As such, greater increases in nutrient delivery are acutely required following concussion to accommodate augmented metabolic demand associated with a cognitive task, potentially stressing the NVC response [11]. Further, previous research has noted that the temporal NVC response is greatest during systole [49], which displayed the greatest reductions in the current investigation (Figure 1). This further supports the notion of assessing cerebrovascular parameters at systole and diastole in addition to the mean. Finally, the biomechanical trauma associated with concussive injuries results in cytoskeletal damage, which may affect the integrity of the neurovascular unit and trigger the release of vasoactive materials (i.e., CO_2_, NO_2_, etc.) [2,3]. As such, it is postulated that disruptions within the NVC response acutely (1-3 days) following concussion observed in the current case may be due to the injury-related ionic, axonal, and cellular perturbations coupled with attenuations in CBF [50].

Across days 1–14 post-concussion, the case in the current investigation experienced a greater degree of sympathetic activation and reduced vagal tone, as indicated by increased heart rate for both sitting and standing paradigms eliciting reductions in HRV metrics, as well as a greater relative LF and subsequent LF/HF ratio (Figure 2 and Figure 3). At rest, the parasympathetic and sympathetic branches of the ANS operate harmoniously to maintain homeostasis through the unconscious regulation of various physiological functions (i.e., heart rate, respiration) [51]. The previous literature has utilized HRV and baroreceptor sensitivity metrics to assess ANS function in both healthy and clinical populations, finding alterations following concussion [14,15,52,53,54]. This may, in part, be the result of the pathophysiological consequences of concussion, including diffuse axonal dysfunction and altered neurotransmission [2,3]. HRV is regulated by output from the brain stem and hypothalamus [55]. As such, concussion-related disruptions within the aforementioned neural regions responsible for regulating ANS function may underpin the increased sympathetic activation and reductions in vagal tone observed in the current investigation. The importance of the ANS function contributing to the regulation of blood pressure variability is highlighted by the existing literature characterizing the modulation of dCA, wherein sympathetic activity is thought to govern cerebral hemodynamic responses at a frequency of 0.10Hz [56]. Thus, it is plausible that ANS impairments may underpin, to a degree, the acute disruptions in dCA observed in the current case report. 

The results demonstrated that dCA gain and normalized gain metrics were elevated across the 14 days in PCA at both 0.05 and 0.10 Hz, while phase reductions were also observed within the MCA at both point-estimates of interest (Figure 4 and Figure 5). Transfer function analysis is often utilized to derive phase and gain metrics, providing insight into the cerebral pressure–flow relationship [57]. Gain characterizes the ratio between the magnitude of input and output parameters, with higher gain metrics demonstrating a greater dampening of blood pressure oscillations [57]. Conversely, reductions in gain metrics indicate a reduction in vascular buffering, characterized by the transfer of lower-amplitude blood pressure fluctuations to the cerebrovasculature [57]. Moreover, the brain acts as a high-pass filter, where higher-frequency blood pressure oscillations (>0.20 Hz) are linearly transferred to the cerebrovascular network, while lower-frequency oscillations are effectively buffered [57]. The results from the current investigation indicated that gain metrics were elevated across the initial 14 days post-injury at both 0.05 and 0.10 Hz, indicating potential disruptions within the typical buffering response of the cerebrovascular network (Figure 4 and Figure 5). Phase represents the time delay between blood pressure oscillations and vascular response, wherein higher phase metrics indicate rapid alteration of the cerebrovascular network in response to fluctuations in blood pressure [57]. Reductions within phase parameters observed in the current investigation are congruent with previous findings of attenuated phase metrics at 0.10 Hz, persisting for 2 weeks post-concussion [5]. Moreover, the current dCA result indicates that the greatest alterations appeared to occur when the cerebrovascular was the most pressure-passive (i.e., during diastole), which is consistent with previous reports in the literature [5]. The observed increases and decreases in phase and gain, respectively, further elucidate the effect of concussion on dCA, providing evidence of post-injury impairments within the buffering capacity of the cerebrovascular network required to regulate CBF across fluctuations in blood pressure.

The collective findings based on previous NVC, HRV, and dCA literature, in addition to this case, hold important clinical ramifications. ANS and dCA perturbations were present across the first 5 days post-injury, which coincided with the days on which the case reported the highest levels of clinical symptoms. While symptoms were lower on days 10 and 14, albeit still above 7 (i.e., a common threshold for indicating recovery from concussion) [19], ANS and dCA perturbations remained. Ultimately, this highlights the importance of athletes progressing through all six stages of the graded return-to-play strategy, which, at the quickest, would occur 6 days post-injury. Nonetheless, for the safety and long-term health of an athlete, this could be modified to ensure athletes return to play at least 7 days following symptom resolution. This would increase the likelihood of physiological recovery occurring before an athlete experiences subsequent subconcussive and/or concussive impacts that compound the physiological alterations from the previous concussion. Clinicians should be cognizant of these findings/suggestions when determining if an athlete should return to play, which may be aided by the Sport Concussion Office Assessment Tools [58,59]. However, further research is a necessity in understanding physiologically relevant tests that are also simple and quick to perform in a clinician’s office. 

The limitations of the current case study include the fact the participant was using medication to treat his bipolar diagnosis. Specifically, valproic acid is known to increase γ-aminobutyric acid (i.e., GABA), which is the main inhibitory neurotransmitter in the brain [60]. Escitalopram, similarly, has the potential to blunt neuronal responses [61], although this is more often reported with single-dose or initial administration. These medications would likely have the greatest impact on the NVC response; however, the AUC30 results in Figure 1 correspond to the 75–80th percentile compared to previously published data [49]. Therefore, as the case used these medications consistently for a couple of years prior to the study, homeostasis of signaling between the neurons and vasculature was likely restored. However, a paucity exists regarding the extent to which various medications impact cerebrovascular function and concussion recovery. Future research into this domain is urgently required.

Another limitation in the current study surrounds the difficulties in defining concussion recovery in individuals with premorbid conditions/diagnoses. One-way recovery is defined as when an individual’s symptom severity score returns to 7 or less for three consecutive days [19]. However, previous research has described that those with ADHD, psychiatric and learning disorders, and migraines in the absence of head trauma exhibited concussion-like symptoms [62,63]. This complicates the idea that a single symptom threshold can be used to define recovery as this is confounded by numerous variables. Another unique aspect of the current study is the fact that the individual had 10 previous concussions. Cook and colleagues [59] noted the mean symptom severity score increased with each successive concussion: 6.9 (no history); 7.2 (one previous concussion); 8.9 (two previous concussions); and 11.9 (three or more previous concussions). It would thus be expected that baseline symptoms would be higher in this case compared to in the general population; however, very limited research regarding the onset of concussion symptoms following successive concussions has been completed for individuals with an extensive concussion history. Ultimately, further research is warranted to consider the accumulation of biopsychosocial aspects contributing to injury outcomes to provide a more comprehensive evaluation of recovery [64]. 

## 5. Conclusions

This case study examined the degree and duration of impairment in ANS function and cerebrovascular regulation across the initial 14 days following concussion in an individual with numerous risk factors associated with protracted recovery. The results demonstrated disruptions in the NVC response, increased sympathetic activation and reduced vagal tone, and attenuated dCA post-injury, with physiological impairments persisting despite the reduction in clinical symptoms. These results warrant the need for future research utilizing serial monitoring to evaluate the immediate and acute effects of concussion on cerebrovascular and autonomic parameters within a larger sample of subjects to validate the degree and duration to which physiological dysfunction may underpin recovery trajectories following concussion, as well as the degree to which physiological parameters are associated with biopsychosocial risk factors. Finally, these findings highlight the importance of completing serial testing once clinical recovery has occurred to better understand the recovery time course of physiological parameters post-concussion.

## Figures and Tables

**Table 1:** Previous history of concussion for the case.

Concussion Number	Date of Injury	Age at Injury	Mechanism of Injury	Duration of Recovery
1	January 1996	7 months	Fall—downstairs	Unknown
2	August 2008	13 years	Fall—longboard	Two days
3	November 2011	16 years	SRC—basketball	Three days
4	January 2012	16 years	SRC—basketball	Two days
5	September 2013	18 years	SRC—basketball	One month
6	October 2013	18 years	SRC—basketball	Seven months
7	June 2014	19 years	MVC—whiplash	Two months
8	September 2016	21 years	Struck to head—weightlifting	One month
9	March 2018	23 years	SRC—basketball	Two days
10	August 2019	24 years	SRC—wakeboarding	One month

Sport-related concussion (SRC); motor vehicle collision (MVC).

**Table 2:** Symptom evaluation using the Sport Concussion Assessment Tool 5 across all testing sessions. Symptoms were taken before and after the experimental protocol. Overall condition was taken via a visual analog scale.

	Baseline	Day 1		Day 2		Day 3		Day 4		Day 5		Day 10		Day 14	
		Pre	Post	Pre	Post	Pre	Post	Pre	Post	Pre	Post	Pre	Post	Pre	Post
Headaches	1	4	5	3	3	3	4	2	3	2	3	1	1	1	1
Pressure in Head	1	4	5	3	3	3	4	2	3	2	3	1	1	1	1
Neck Pain	0	1	1	1	1	1	1	1	1	0	0	0	0	0	0
Nausea or Vomiting	0	0	0	0	0	0	0	0	0	0	0	0	0	0	0
Dizziness	0	0	0	0	0	1	0	0	0	0	0	0	0	0	0
Blurred or Double Vision	0	0	0	0	0	0	0	0	0	0	0	0	0	0	0
Balance Problems	0	0	0	0	0	0	0	0	0	0	0	0	0	0	0
Sensitivity to Light	1	2	2	2	1	2	2	1	0	1	1	0	0	1	0
Sensitivity to Noise	0	2	2	2	1	2	2	1	0	1	1	0	0	1	0
Feeling Slowed Down	1	1	1	1	1	1	1	1	2	1	1	0	0	1	0
Feeling “in a Fog”	0	1	0	1	1	1	2	1	1	1	1	0	0	0	0
Don’t Feel Right	0	0	0	1	1	1	1	1	1	1	1	1	1	1	1
Difficulty Concentrating	0	2	2	1	1	0	0	0	0	0	0	0	0	0	0
Difficulty Remembering	0	2	2	1	1	0	0	0	0	0	0	0	0	0	0
Fatigue or Low Energy	1	2	1	1	2	3	3	2	1	2	2	1	1	0	1
Confusion	0	0	0	0	0	1	1	1	0	1	0	0	0	0	0
Drowsiness	0	2	2	0	2	2	1	1	1	2	1	1	0	0	0
More Emotional	1	3	2	2	2	3	2	3	3	3	1	1	1	0	0
Irritability	0	3	4	3	2	3	2	3	3	3	2	1	1	0	0
Sadness	1	2	1	2	2	3	2	2	3	3	1	1	0	0	0
Nervous or Anxious	0	0	0	1	1	2	1	1	1	2	1	0	0	0	0
Trouble Falling Asleep	0	0	0	2	1	2	2	1	1	2	2	1	1	1	1
**Total Symptom Score**	7	14	13	16	17	17	16	16	13	15	14	9	7	7	5
**Symptom Severity Score**	7	31	30	27	26	34	31	24	24	27	21	8	7	7	5
**Overall Condition**	0	4	7	4	7	5	8	4	7	3	5	2	2	1	1
**Post 30 Overall Condition**			5		5		7		2		2		1		1

Note: The symptoms that were elevated to the greatest extent during the first five days post-injury were headaches, pressure in head, fatigue, emotionality, irritability, and sadness.

## Data Availability

The materials and data that support the findings of this study are available from the corresponding author (J.S.B.) upon reasonable request.
